# METTL16 inhibits pancreatic cancer proliferation and metastasis by promoting MROH8 RNA stability and inhibiting CAPN2 expression – experimental studies

**DOI:** 10.1097/JS9.0000000000002116

**Published:** 2024-10-22

**Authors:** Tingzhuang Yi, Chunming Wang, Xia Ye, Jie Lin, Cheng Lin, Fengzhen Qin, Wanlin Yang, Yulu Ye, Dengchong Ning, Jinyan Lan, Huafu Li, Chunying Luo, Jian Ma, Zhongheng Wei

**Affiliations:** a Department of Oncology, Affiliated Hospital of YouJiang Medical University for Nationalities/Key Laboratory of Molecular Pathology in Tumors of Guangxi Higher Education Institutions, Baise, Guangxi, People’s Republic of China; b Department of Hepatopancreatobiliary & Vascular Surgery, The Second Affiliated Hospital of Guangxi Medical University, Nanning, People’s Republic of China; c Youjiang Medical University for Nationalities, Baise, Guangxi, People’s Republic of China; d Affiliated Hospital of Youjiang Medical University for Nationalities, Baise, Guangxi, People’s Republic of China; e Imperial College London, Sir Alexander Fleming Building, South Kensington Campus, London, UK; f Department of Hepatobiliary Surgery, Jining Public Health Medical Center, Jining, People’s Republic of China

**Keywords:** Pancreatic cancer, m6A, METTL16, TBP, MROH8, CAPN2

## Abstract

**Background:**

N6-methyladenosine (m6A) modification plays a crucial role in the progression of various cancers, including pancreatic cancer, by regulating gene expression. However, the specific mechanisms by which m6A affects pancreatic cancer metastasis remain unclear. This study aims to elucidate the role of METTL16, an m6A writer gene, in regulating core genes such as CAPN2 and MROH8, influencing tumor growth and metastasis.

**Materials and Methods:**

Transcriptomic data from pancreatic cancer patients in The Cancer Genome Atlas (TCGA) were analyzed to identify m6A-related genes. We performed correlation and survival analyses to uncover core genes influenced by m6A expression. Functional assays, including METTL16 knockdown and overexpression experiments, were conducted in pancreatic cancer cell lines, patient-derived organoids, and animal models. Immunofluorescence, co-immunoprecipitation (Co-IP), and m6A-specific quantitative PCR were used to validate protein interactions and m6A modifications. Chromatin immunoprecipitation (ChIP) analysis was utilized to investigate transcription factor binding at gene promoter regions.

**Results:**

METTL16 and METTL3 were identified as key m6A regulators associated with improved prognosis in pancreatic cancer patients (*P*<0.05). CAPN2, CHMP2B, ITGA3, ITGA6, ITPR1, and RAC1 were identified as core genes linked to m6A expression, all significantly correlated with patient prognosis (*P*<0.05). METTL16 overexpression significantly inhibited tumor growth and metastasis (*P*<0.001) by downregulating CAPN2 through an indirect mechanism involving the transcription factor TBP and the gene MROH8. MROH8 negatively regulated CAPN2 by promoting TBP degradation, with METTL16 enhancing MROH8 mRNA stability through m6A modifications (*P*<0.01). Functional assays demonstrated that METTL16 and YTHDC2 (an m6A reader) collaboratively enhanced MROH8 mRNA stability, thereby inhibiting CAPN2 expression and reducing tumor proliferation and metastasis (*P*<0.001).

**Conclusion:**

This study reveals a novel regulatory axis involving METTL16, MROH8, and TBP that modulates CAPN2 expression, contributing to the suppression of pancreatic cancer progression. The METTL16–MROH8–TBP–CAPN2 pathway offers potential therapeutic targets for pancreatic cancer treatment, highlighting the significance of m6A modifications in tumor regulation. Further clinical validation is needed to confirm these findings in human patients.

## Introduction

Highlights

- METTL16, an important m6A writer gene, promotes the translation of MROH8 by regulating its mRNA stability. Subsequently, MROH8 binds to the TBP protein and promotes TBP degradation.
- TBP, in turn, binds to the promoter of CAPN2, enhancing CAPN2 translation levels. Finally, CAPN2 significantly promotes the proliferation and metastasis of pancreatic cancer cells.
- m6A in pancreatic cancer may help improve patient survival.

Ductal adenocarcinoma of the pancreas remains one of the deadliest malignancies, with an estimated 5-year survival rate of only 3% for patients diagnosed with metastatic diseases^1^. Surgical resection is the only effective treatment option. However, more than 80% of patients are diagnosed with pancreatic cancer when their disease has progressed beyond operable limits^2^. Although numerous genetic studies have identified many alterations in key genes, the molecular mechanisms of pancreatic cancer development remain to be fully understood^3^. Current research primarily focuses on identifying genetic and epigenetic alterations that contribute to pancreatic cancer progression. However, the intricate regulatory networks, particularly those involving post-transcriptional modifications such as m6A methylation, are still not fully delineated. This study addresses these gaps by investigating the role of m6A modifications, especially the m6A writer gene METTL16, in regulating pancreatic cancer metastasis. Therefore, addressing the problem of pancreatic cancer metastasis, such as by discovering the molecular mechanisms that regulate the growth or spread of pancreatic cancer cells, could significantly improve patient outcomes and potentially reverse the high mortality rate associated with this disease.

As the most prevalent gene regulatory mechanisms, alternative splicing (AS) and N6-methyladenosine (m6A) have become significant genetic modifications in human cancers^4^. m6A has been detected on adenosine within the consensus sequence G [G > A] m6AC [U > A > C] in various mRNA transcripts^5^. Notably, m6A is a dynamic modification induced by methyltransferase complexes containing METTL3, METTL14, and other regulatory subunits and removed by RNA demethylases FTO and ALKBH5^6^. Although numerous m6A-related studies have shown a close link between the known writer and eraser genes in regulating pancreatic cancer growth and metastasis, the m6A regulatory system is highly complex, and our understanding of its intricate network remains limited^3^.

This study aims to build upon the current understanding by focusing on the unexplored role of METTL16 in pancreatic cancer. Specifically, we aim to investigate how METTL16, an m6A writer gene, interacts with key downstream targets, such as CAPN2 and MROH8, to influence cancer metastasis. We analyzed The Cancer Genome Atlas (TCGA) data to explore the correlation between known m6A genes and other genes involved in expression. We aimed to identify m6A-regulated genes that influence patient prognosis by comparing the prognostic relevance of these correlated genes. Upon comparing the expression of m6A writer genes with patient prognosis, we were surprised to find that the expression of METTL16 and METTL3 was associated with better patient outcomes. This finding contrasts with observations in other tumors^7^.

It is understood that m6A modifications do not directly regulate tumors but influence tumor growth and metastasis by affecting the mRNA of target genes. Our analysis revealed that CAPN2 expression is closely related to METTL16 expression, but they are regulated through a negative feedback mechanism, which is contrary to the known m6A regulatory mechanisms. This initially posed a challenge for our study. However, further analysis led to an unexpected discovery. We hypothesized that METTL16 does not directly regulate CAPN2 but functions by regulating MROH8, a gene previously uncharacterized in the context of m6A modifications. Subsequent experiments confirmed this hypothesis; we found that METTL16 inhibits CAPN2 expression by regulating MROH8, which in turn affects the progression and metastasis of pancreatic cancer. This study thus proposes a novel regulatory axis involving METTL16, MROH8, and CAPN2, expanding our understanding of how m6A modifications influence pancreatic cancer progression and offering potential new therapeutic targets.

## Method

**Figure 1:**
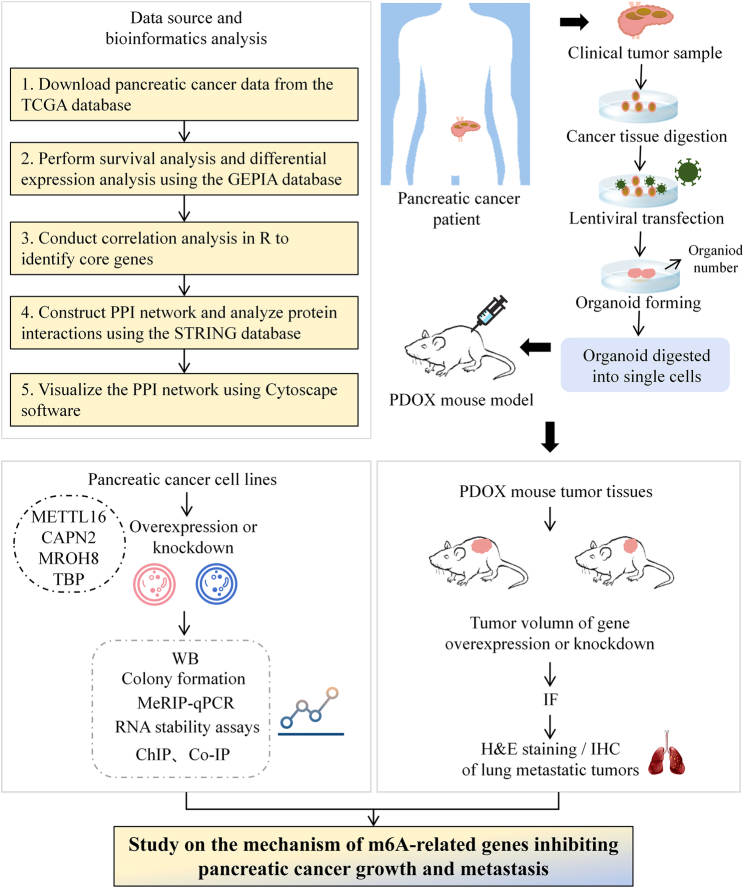
Research flowchart. ChIP, chromatin immunoprecipitation; Co-IP, co-immunoprecipitation; GEPIA, gene expression profiling interactive analysis; H&E, hematoxylin and eosin; IF, immunofluorescence; IHC, immunohistochemistry; MeRIP-qPCR, methylated RNA immunoprecipitation followed by quantitative polymerase chain reaction; PDOX, patient-derived orthotopic xenograft; PPI, protein–protein interaction; TCGA: The Cancer Genome Atlas; WB, Western blot.

This study first conducted bioinformatics analysis using data from the TCGA database to identify genes associated with the prognosis of pancreatic cancer. Subsequently, clinical pancreatic cancer samples were collected, and gene overexpression and knockout experiments were performed in various pancreatic cancer cell lines to investigate the effects of these genes on tumor growth. The in-vivo effects of these genes were further validated using the PDOX mouse model. Molecular biology techniques were employed to explore the regulatory mechanisms between genes, particularly focusing on the roles of METTL16 and CAPN2 in pancreatic cancer. Together, these methods revealed the regulatory networks and potential mechanisms by which these genes influence pancreatic cancer progression (Fig. 1).

### Data source and bioinformatics analysis

Transcript fragment per kilobase (FPKM)-normalized transcript RNA-sequencing data and relevant clinical information were downloaded from the TCGA database (https://portal.gdc.cancer.gov/) (pancreatic cancer dataset). Each corresponding gene was analyzed using the GEPIA database (GEPIA2) for survival analysis and differential gene comparisons with normal tissues. The focus was on m6A ‘writer’ genes, including METTL3, METTL14, METTL16, WTAP, VIRMA, ZC3H13, RBM15, and RBM15B, to evaluate their prognostic significance in pancreatic cancer patients. Differential gene expression of m6A and other mRNAs was analyzed using correlation analyses in the R language, and core genes associated with m6A expression. The protein–protein interaction (PPI) network was generated using the STRING database with a minimum interaction score threshold of low confidence (0.4) and was visualized through Cytoscape software, version 3.8.2^8,9^.

### Clinical sample collection

With the approval of the Ethics Committee of Baise People’s Hospital, three pairs of clinical samples were collected from pancreatic cancer patients for this study. After rapid freezing with liquid nitrogen, all tissues were stored at −80°C until use. Each patient participating in this study did not receive any other treatments and signed a written informed consent before the procedure.

### Cell culture

Human pancreatic cancer cell lines (ASPC1, BXPC2, CAPAN1, CAPAN2, and CFPAC1) and human embryonic kidney cell line (HEK293T) were obtained from the American Type Culture Collection (ATCC; Manassas, VA, USA). The human normal pancreatic cell line (HPDE6c7) was derived from MERCK. THLE-3 cells were cultured in Bronchial Epithelial Growth Medium (BEGM) medium (LONZA, Basel, Switzerland), and the cells were cultured in Dulbecco’s modified Eagle’s medium (DMEM; GIBCO, Rockville, MD, USA) with 10% FBS plus 1% penicillin/streptomycin (Thermo Fisher Scientific). All media were used for cell culture at 37°C with 5% CO_2_. The cell experiments were divided into four groups: control group (con), gene overexpression group, gene knockout (knockdown) group, and negative control group (sh-con). The genes studied in these groups included METTL16, CAPN2, MROH8, and TBP.

### Cell transfection

The following constructs were purchased from RiboBio (Guangzhou, China): pcDNA empty vector, pcDNA-CAPN2, pcDNA-METTL16, pcDNA-ythdc2, pcDNA-MROH8-flag, pcDNA-TBP-gfp, shRNA Normal Control (sh-con), and shRNA targeting the above genes. The transfection process was performed using Lipofectamine 2000 (Invitrogen, Carlsbad, CA, USA) or X-tremeGENE transfection reagent (Thermo Fisher Scientific, USA) according to the manufacturer’s protocol^10^. Each transfection experiment was conducted in the aforementioned four groups, with cells in the gene overexpression group transfected with constructs such as pcDNA-CAPN2, pcDNA-METTL16, etc., and the gene knockout (knockdown) group transfected with corresponding shRNAs targeting these genes.

### RT-PCR (real-time PCR)

Total RNA was isolated from cells using TRIzol reagent (Invitrogen, Carlsbad, CA, USA) according to the manufacturer’s protocol. Subsequently, cDNA was synthesized from 1 μg of RNA using the Qiagen kit (Qiagen, Valencia, CA, USA). Real-time PCR was performed using the SYBR Premix Ex Taq kit (Takara, Otsu, Japan) on a StepOnePlus Real-Time PCR system (Applied Biosystems, Shanghai, China). The relative expression was quantified using the 2^−ΔΔCt^ method with GAPDH as an endogenous control^9^. The primers used in this study are listed in Table 1.

**Table 1:** The primers used in this study.

Gene name	Sequence	
	Forward sequence	Reverse sequence
METTL16	TGGAGCAACCTTGAATGGCTGG	CCATCAGGAGTGTCTTCTGTGG
METTL3	CTATCTCCTGGCACTCGCAAGA	GCTTGAACCGTGCAACCACATC
CAPN2	AGGACATGCACACCATCGGCTT	CGGAGGTTGATGAAGGTGTCTG
ITGA6	CGAAACCAAGGTTCTGAGCCCA	CTTGGATCTCCACTGAGGCAGT
ITPR1	GTGACAGGAAACATGCAGACTCG	CAGCAGTTGCACAAAGACAGGC
RAC1	CGGTGAATCTGGGCTTATGGGA	GGAGGTTATATCCTTACCGTACG
ITGA3	GCCTGACAACAAGTGTGAGAGC	GGTGTTCGTCACGTTGATGCTC
CHMP2B	GGCTATAATCAGAGATCGAGCAG	CTCGTCTTCTGTTTCCGTAGATG
MROH8	CAGCCTAGAGTCCGCCAACAAA	ACCGACAGCATTCTCCACACCT
YTHDC2	GAAAGCTCCTGAACCTCCACCA	GGTTCTACTGGCAAGTCAGCCA
TBP	TGTATCCACAGTGAATCTTGGTTG	GGTTCGTGGCTCTCTTATCCTC

### Colony formation assay

Transfected cells were seeded into 6-well plates (Thermo Fisher Scientific) and cultured for approximately 2 weeks for the colony formation assay. Subsequently, they were fixed with methanol and stained with crystal violet. Stained colonies (containing more than 50 cells) were manually counted. This experiment was performed three times.

### Human organoids

Organoid generation was performed as follows: Pancreatic cancer samples were placed in ice-cold G solution containing 50 μg/ml penicillin–streptomycin (Thermo Fisher Scientific), chopped on ice, and incubated in DMEM containing 1 mg/ml collagenase V (Sigma-Aldrich) for 1 h at 37°C. Digestion was stopped by adding ice-cold PBS, followed by centrifugation at 4°C (300 G, 5 min). Further digestion was carried out with TrypLE (Thermo Fisher Scientific) for 5 min at 37°C and then stopped with a large amount of PBS. The suspension was filtered through a 40 μm nylon mesh, centrifuged, and the cells were resuspended in culture medium. The organoids were passaged with TrypLE every 2 weeks. The medium for establishing and culturing human pancreatic cancer-like organs was as described in the literature^11^.

### The patient-derived orthotopic xenograft (PDOX) mouse model

Eight-week-old BALB/C NUDE mice (002019; Jackson Laboratory) were housed under standard conditions with controlled temperatures (20–26°C), light/dark cycle (12 h/12 h), and provided with drinking water and standard pellet diets. The mice were divided into four groups: control, gene overexpression, gene knockout, and negative control. Organoids were transduced with lentiviral particles to overexpress METTL16, CAPN2, MROH8-flag, or TBP-gfp, or with lentivirus encoding shRNA to knock down METTL16, CAPN2, MROH8, or TBP. Control groups received unmodified organoids or organoids transduced with empty vectors (RiboBio, Guangzhou, China). Organoid transduction was performed with lentivirus at a multiplicity of infection (MOI) of 10, using 8 μg/ml polybrene. After 24 h of incubation, the medium was replaced, and organoids were cultured for an additional 48–72 h^12^. The organoids were digested into single cells with TrypLE and counted, and 100 000 cells were mixed with a 50% Matrigel/50% Hank’s balanced salt solution (HBSS) and subcutaneously inoculated into BALB/C NUDE mice (six mice per group). The mice were euthanized after 1 month, and the tumors were excised. All tumors were photographed, and their mass and volume were determined using the formula: Tumor volume (mm^3^) = 0.5 × width^2^ × length^13^. In-vivo experiments were performed according to the Institutional Animal Care and Use Committee (IACUC). The experimental protocol was approved by the Clinical Research and Animal Experimentation Ethics Committee of the Affiliated Hospital of Youjiang Medical College of Nationalities]. Work has been reported in accordance with the ARRIVE guidelines, Supplemental Digital Content 1, http://links.lww.com/JS9/D516 (Animals in Research: Reporting In Vivo Experiments)^14^.

### MeRIP-qPCR

The m6A immunoprecipitation (MeRIP) assay was performed using the Magna MeRIP m6A kit (Millipore, Germany) following the manufacturer’s instructions. Pancreatic cancer cell lines were lysed, and total RNA was extracted, digested with DNase I, and fragmented to ~100 nucleotides. RNA was immunoprecipitated with 12 μg of anti-m6A antibody pre-incubated with magnetic beads. After incubation, the RNA-bead complex was washed, and bound RNA was purified using phenol-chloroform extraction. Purified RNA was analyzed by qRT-PCR to determine m6A enrichment at target sites, with primers designed to cover predicted m6A modification regions. Input RNA was used for normalization in qRT-PCR analysis^15^.

### RNA stability assays

Cells were treated with 2.5 μg/ml actinomycin D (MedChemExpress, Shanghai, China) for 0, 2, 4, 6, and 8 h. Subsequently, total RNA was isolated from these cells treated with actinomycin D using AG RNAex Pro Reagent, normalized to GAPDH, and subjected to RT-PCR analysis. The relative mRNA expression levels at each time point were normalized to the 0-h control. The remaining mRNA levels were logarithmically transformed (natural logarithm), and linear regression analysis was performed by plotting the log-transformed mRNA levels against time. The decay rate constant (*k*) was determined from the slope of the regression line, and the half-life (*t*_1/2_) of the mRNA was calculated using the formula: *t*_1/2_=*ln*_2_/−*k*. The calculation method is based on previously reported methods in the literature^16^.

### Immunohistochemical staining (IHC)

Tumor tissues from the PDOX mouse model were collected, fixed in 10% neutral buffered formalin (NBF, Sigma) for 16 h, dehydrated with 70% ethanol, and embedded in 4 μm paraffin sections. Hematoxylin and eosin (H&E) staining was performed according to standard procedures^17^. Immunohistochemical analysis was performed to detect the expression levels of Ki67 as a marker for cell proliferation and CAPN2 to assess its role in tumor progression. After heat-mediated antigen retrieval in 10 mM sodium citrate buffer (pH 6.2), endogenous peroxidase was blocked with 1.6% hydrogen peroxide and stained with DAB according to the manufacturer’s instructions for Ki67 Rabbit Polyclonal Antibody (PTG, 27309-1-AP), and CAPN2 Polyclonal Antibody (PTG, 11472-1-AP). Five fields of view per slide were randomly selected to count positive cells.

### Immunofluorescent staining (IF)

Immunofluorescence staining was used to assess the colocalization and expression levels of CAPN2, METTL16, and Ki67 in pancreatic cancer cell lines and PDOX mouse tumor tissues, focusing on their potential interaction and contribution to cell proliferation and tumor progression. Cells or organoids were grown in glass-bottomed tissue culture plates (Ibidi, 191218/2), fixed with 5% NBF for 10 min, and blocked with PBS containing 10% FCS, 1% BSA (Sigma), and 0.2% Triton-X. For tumor tissues, 4 μm paraffin-embedded sections from PDOX mice were prepared and processed similarly. Primary antibodies were incubated in a blocking buffer at 4°C for 16 h, and fluorescent secondary antibodies with 3 μM DAPI were incubated in a blocking buffer at 20°C for 1–6 h. Fluorescent staining was imaged on a Zeiss LSM 780 confocal microscope. Detailed tissue preparations were immunohistochemically stained. Secondary antibodies were conjugated with fluorescent moieties and incubated with 3 μM DAPI in the dark. Prior to mounting, slides were incubated in 0.1% (w/v) Sudan Black B (Sigma) and 70% ethanol to reduce the background signal. Antibodies used included CAPN2 Monoclonal Antibody (ptglab, 66977-1-Ig), METTL16 Polyclonal Antibody (PTG, 19924-1-AP), and Ki67 Rabbit Polyclonal Antibody (PTG, 27309-1-AP).

### Immunoprecipitation (ChIP)

ChIP experiments were performed to verify the direct relationship between TBP and CAPN2, as recommended by the manufacturer (Beyotime). After fixation of the cells in 1% formaldehyde and incubation with glycine solution, the cells were washed with PBS containing protease and phosphatase inhibitors (Beyotime), lysed (0°C; 10 min) with SDS lysis buffer, and the DNA fragments were sonicated. The sonicated chromatin was confirmed to have an average length of 200–500 bp by agarose gel electrophoresis. Immunoprecipitation was performed using 5 μg of anti-TBP rabbit antibody (#44059, Cell Signaling Technology) or IgG control overnight at 4°C with 50 μl of Protein A/G beads. Beads were harvested using a magnetic separator, and DNA was purified using a purification kit. qpCR was performed to detect enrichment of the CAPN2 promoter.

### Western blot

Transfected cells were lysed using radioimmunoprecipitation assay (RIPA) lysis buffer (CST, Danvers, MA, USA) supplemented with protease and phosphatase inhibitors. After centrifugation for 30 min, protein concentrations were determined using the Pierce Bicinchoninic Acid (BCA) Protein Detection Kit (Bio-Rad Laboratories, Hercules, CA, USA). Proteins in each sample were separated by SDS-polyacrylamide gel electrophoresis (SDS-PAGE) and subsequently transferred to polyvinylidene difluoride membranes (PVDF; Bio-Rad Laboratories, Hercules, CA, USA). Twenty micrograms of protein from each sample were loaded onto SDS-polyacrylamide gel electrophoresis (SDS-PAGE) gels for separation. Proteins were subsequently transferred to polyvinylidene difluoride (PVDF) membranes (Bio-Rad Laboratories, Hercules, CA, USA) using a wet transfer system at 4°C for 90 min. The membranes were incubated overnight at 4°C with 5% skimmed milk, followed by primary antibodies: CAPN2 Monoclonal Antibody (ptglab, 66977-1-Ig), METTL16 Polyclonal Antibody (PTG, 19924-1-AP), C20orf132 Polyclonal Antibody (Invitrogen, PA5-63836), TBP Monoclonal Antibody (ptgcn, 66166-1-Ig), GFP Polyclonal Antibody (Thermo Fisher, #A10262), and Monoclonal ANTI-FLAG M2 Antibody (Sigma-Aldrich, F3165-.2MG). This was followed by incubation with a secondary antibody (ab205718, 1/10000; Abcam) for 1 h at room temperature using enhanced chemiluminescence (ECL; US Microwells). GAPDH or β-actin served as internal controls. After exposure, the protein bands were quantified using ImageJ software for densitometry analysis. Each experiment was repeated three times.

### Immunoprecipitation (IP) and Co-immunoprecipitation (Co-IP) assay

For the IP and CO-IP assay, cell lysates were extracted using non-denaturing methods following the instructions provided with the IP/CO-IP extraction kit (Abbkine). The lysates were pre-cleared with 20 μl of protein A/G agarose beads to reduce non-specific binding before antibody incubation. Lysates were incubated with mouse anti-GFP (# MA5-15256, Thermo Fisher), microspheres coupled with rabbit anti-FLAG (ab236777, Abcam), or rabbit IgG overnight at 4°C. The immunoprecipitated complexes were washed four times with cold lysis buffer to remove non-specifically bound proteins, and then the proteins were eluted by boiling in SDS sample buffer for 5 min. After washing, a Western blot was performed by boiling in SDS sample buffer, and the secondary antibodies used were HRP-conjugated donkey anti-rabbit IgG LCS (1:1000) and HRP-conjugated donkey anti-mouse IgG LCS (1:1000).

### LC–MS/MS analysis

For LC–MS/MS analysis, each sample (8 μl) was treated with High-Select Top14 Abundant Protein Depletion Resin following the manufacturer’s instructions (A36372, Thermo). Following depletion, proteins were analyzed by LC–MS/MS using an Easy-nLC 1200 system and a Q Exactive HF-X hybrid quadrupole-Orbitrap mass spectrometer (Thermo Fisher Scientific, San Jose, USA). Raw data were acquired using data-independent acquisition methods and analyzed with Spectronaut 15.0 software (Biognosys AG, Switzerland). The *Q* value (false discovery rate, FDR) threshold was set to 1% at both the peptide and protein levels. The average of the first three filtered peptides truncated by a 1% *Q* value was used to determine the number of major groups.

### Statistical analysis

All data are presented as mean ± standard deviation, and a *P* value less than 0.05 was considered statistically significant. Kaplan–Meier survival analysis was conducted, and log-rank tests were used to compare survival curves. For comparisons between multiple groups, a two-way analysis of variance (ANOVA) was used, followed by appropriate post-hoc tests (Bonferroni or Tukey’s test) for pairwise comparisons. For comparisons between two groups, Student’s *t*-test was applied for normally distributed data, while the Mann–Whitney *U* test was used for non-normally distributed data. All statistical analyses were conducted using SPSS 18.0.

## Result

### Exploring the identification and validation of core genes correlated with m6A expression

**Figure 2:**
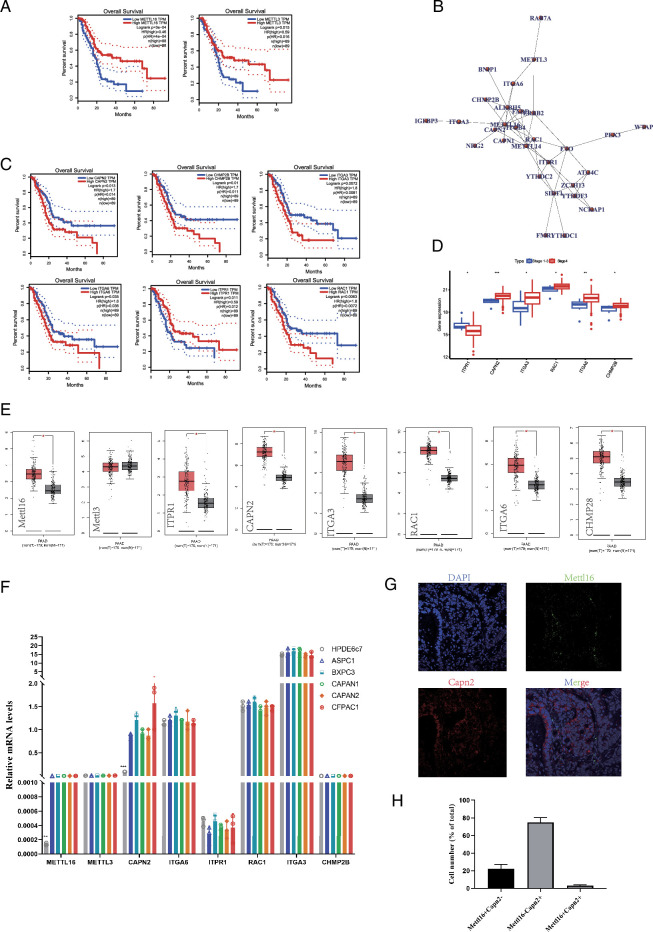
METTL16 and CAPN2 can interact and influence the prognosis of pancreatic cancer patients. (A) The left panel shows the high and low expression of the m6A writer gene METTL16 and its association with patient prognosis in the TCGA database. The right panel shows the high and low expression of the m6A writer gene METTL3 and its association with patient prognosis in the TCGA database. (B) Protein interaction plots show the correlation analysis between m6A-related genes and the expression levels of other mRNAs. (C) High and low expression of CAPN2, CHPM2B, ITGA3, ITGA8, ITPR1, and RAC1 in the TCGA database and their association with patient prognosis. (D) Comparison of the expression levels of CAPN2, CHPM2B, ITGA3, ITGA8, ITPR1, and RAC1 across stages 1, 2, 3, and 4 in the TCGA database. (E), Comparison of the expression levels of METTL16, METTL3, CAPN2, CHPM2B, ITGA3, ITGA8, ITPR1, and RAC1 in paraneoplastic and carcinoma tissues in the TCGA database. (F), RT-qPCR comparison of METTL16, METTL3, CAPN2, CHPM2B, ITGA3, ITGA8, ITPR1, and RAC1 expression levels in cell lines HPDE6c7, ASPC1, BXPC3, CAPAN1, CAPAN2, and CFPAC1. (G) Representative images of immunofluorescence showing METTL16 and CAPN2 expression in pancreatic cancer patients. (H) Statistical analysis of positive cells from panel G.

In our previous discussions, we highlighted the close association between m6A and the development of pancreatic cancer. To deepen our understanding, we aimed to elucidate which m6A genes regulate core genes. We analyzed transcriptomic data from TCGA pancreatic cancer patients. Our initial focus was on identifying m6A ‘writer’ genes that influence the prognosis of these patients. Specifically, we investigated whether METTL3, METTL14, METTL16, WTAP, VIRMA, ZC3H13, RBM15, and RBM15B were associated with patient prognosis. Surprisingly, we found that only METTL3 and METTL16 were significantly correlated with a better prognosis (Fig. 2A) (*P*<0.05). Next, we explored the genes associated with m6A gene expression through correlation analysis. By correlating these genes with patient prognosis, we identified CAPN2, CHMP2B, ITGA3, ITGA6, ITPR1, and RAC1 as core genes associated with m6A expression, all of which were correlated with patient prognosis (Fig. 2B, C). Furthermore, these genes showed significantly higher expression in patients at advanced stages (*P*<0.05) (Fig. 2D). To determine if these genes were specific targets in tumors, we compared their expression levels between tumor and non-tumor samples in the TCGA database. We observed significantly higher expression of METTL16, ITPR1, CAPN2, ITGA3, RAC1, ITGA6, and CHMP28 in tumor samples (*P*<0.05) (Fig. 2E). Given that the TCGA database includes non-tumor cells, we further compared the expression of these genes in tumor and non-tumor cells. Analyzing mRNA profiles of different pancreatic cancer cell lines and normal pancreatic cell lines, we found that CAPN2 was upregulated in tumor cell lines compared to normal pancreatic cell lines (Fig. 2F). Moreover, PPI analysis results indicated an interaction between METTL16 and CAPN2 (Fig. 2B). To verify this negative correlation, we conducted immunofluorescence analysis of clinical specimens, confirming the relationship between METTL16 and CAPN2 (Fig. 2G, H).

### METTL16 overexpression significantly inhibits pancreatic cancer tumor growth

**Figure 3:**
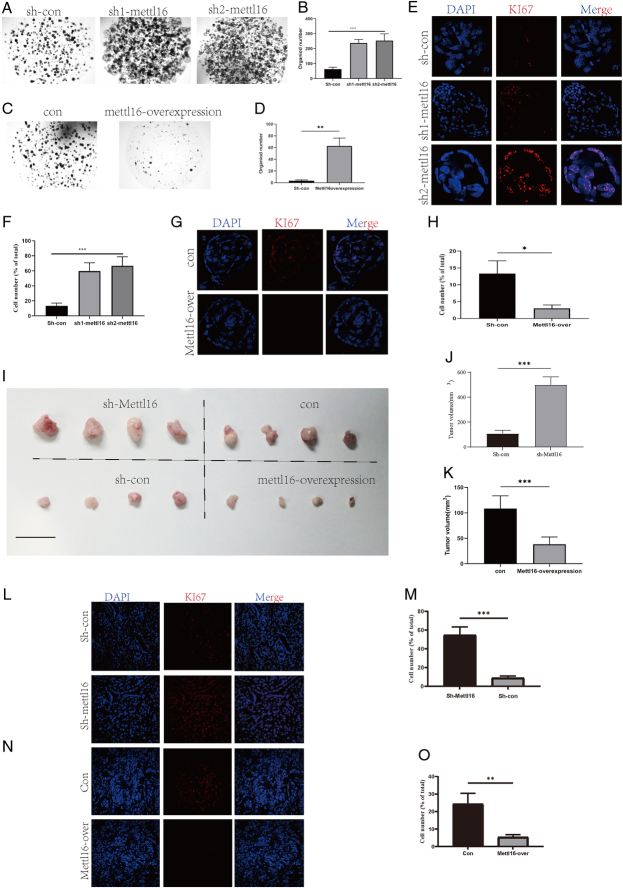
METTL16 suppresses the proliferation of human pancreatic cancer organoids and subcutaneous tumors. (A) Representative images showing the effect of METTL16 knockdown on human pancreatic cancer organoid formation. (B) Bar graph showing the quantification of organoid formation in panel A. (C) Representative images showing the effect of METTL16 overexpression on human pancreatic cancer organoid formation. (D) Bar graph showing the quantification of organoid formation in panel C. (E) Representative immunofluorescence images showing the effect of METTL16 knockdown on KI67 expression in human pancreatic cancer organoids. (F) Bar graph showing the quantification of KI67 expression in panel E. (G) Representative immunofluorescence images showing the effect of METTL16 overexpression on KI67 expression in human pancreatic cancer organoids. (H) Bar graph showing the quantification of KI67 expression in panel G. (I) Representative images showing tumor size following subcutaneous injection of human pancreatic cancer organoids with METTL16 knockdown or overexpression. (J) Bar graph showing tumor size quantification after METTL16 knockdown from panel I. (K) Bar graph showing tumor size quantification after METTL16 overexpression from panel I. (L) Representative immunofluorescence images showing the effect of METTL16 knockdown on KI67 expression in subcutaneous tumors derived from human pancreatic cancer organoids. (M) Bar graph showing the quantification of KI67 expression in panel L. (N) Representative immunofluorescence images showing the effect of METTL16 overexpression on KI67 expression in subcutaneous tumors derived from human pancreatic cancer organoids. (O) Bar graph showing the quantification of KI67 expression in panel N. ^*^ *P*<0.05, ^**^ *P*<0.01, ^***^ *P*<0.001.

Based on our previous data, we hypothesized that the m6A gene METTL16 inhibits CAPN2 by suppressing its expression. We also observed a significant correlation between METTL16 and poor patient prognosis. To investigate how METTL16 affects tumor cell growth, we conducted experiments using METTL16 knockdown human pancreatic cancer-like organoids and pancreatic cancer cell lines (Supplementary Fig. S1A, B, Supplemental Digital Content 2, http://links.lww.com/JS9/D517). Remarkably, our knockdown of METTL16 significantly promoted tumor cell growth in both organoid (Fig. 3A, B) and pancreatic cancer cell lines (*P*<0.01) (Supplementary Fig. S1C, D, Supplemental Digital Content 2, http://links.lww.com/JS9/D517). Conversely, when we overexpressed METTL16 (Supplementary Fig. S1E, F, Supplemental Digital Content 2, http://links.lww.com/JS9/D517), we observed a significant inhibition of tumor growth (Fig. 3C, D and Supplementary Fig. S1G, H, Supplemental Digital Content 2, http://links.lww.com/JS9/D517). This effect was attributed to its impact on cell proliferation (Fig. 3E, F, G, H). To further validate these findings in vivo, we conducted nude mice subcutaneous tumor-forming experiments. As suspected, METTL16 knockdown significantly promoted tumor growth (*P*<0.001) (Fig. 3I, J), while METTL16 overexpression significantly inhibited tumor growth (*P*<0.001) (Fig. 3I, K). Additionally, immunofluorescence Ki67 staining of the obtained tumors revealed a significant increase in Ki67-positive cells after METTL16 knockdown (*P*<0.001) (Fig. 3L, M) and a significant decrease after overexpression (*P*<0.01) (Fig. 3N, O).

### CAPN2 overexpression significantly promotes pancreatic cancer tumor growth

**Figure 4:**
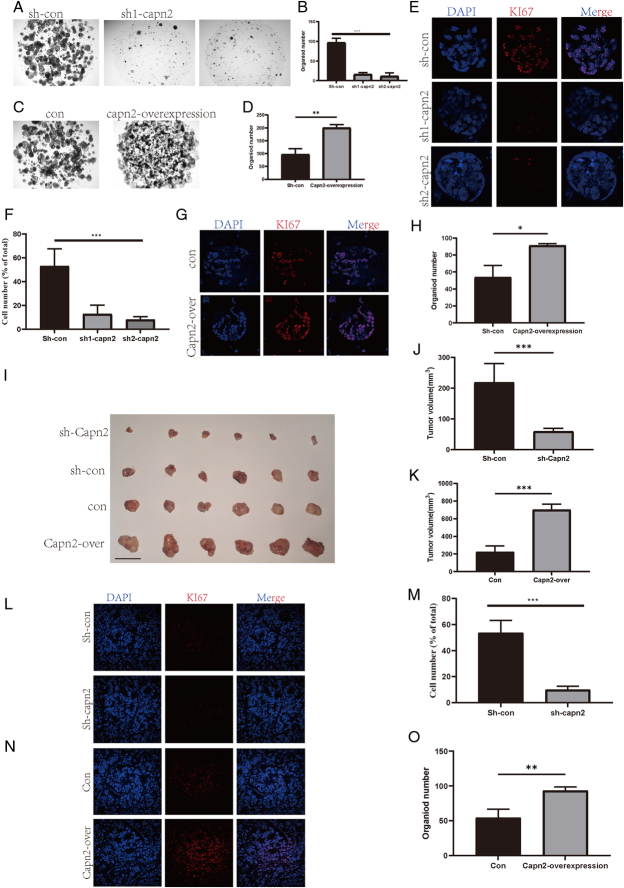
Capn2 promotes the proliferation of human pancreatic cancer organoids and subcutaneous tumors. (A) Representative images showing the effect of Capn2 knockdown on human pancreatic cancer organoid formation. (B) Bar graph showing the quantification of organoid formation in panel A. (C) Representative images showing the effect of Capn2 overexpression on human pancreatic cancer organoid formation. (D) Bar graph showing the quantification of organoid formation in panel C. (E) Representative immunofluorescence images showing the effect of Capn2 knockdown on KI67 expression in human pancreatic cancer organoids. (F) Bar graph showing the quantification of KI67 expression in panel E. (G) Representative immunofluorescence images showing the effect of Capn2 overexpression on KI67 expression in human pancreatic cancer organoids. (H) Bar graph showing the quantification of KI67 expression in panel G. (I) Representative images showing tumor size following subcutaneous injection of human pancreatic cancer organoids with Capn2 knockdown or overexpression. (J) Bar graph showing tumor size quantification after Capn2 knockdown from panel I. (K) Bar graph showing tumor size quantification after Capn2 overexpression from panel I. (L) Representative immunofluorescence images showing the effect of Capn2 knockdown on KI67 expression in subcutaneous tumors derived from human pancreatic cancer organoids. (M) Bar graph showing the quantification of KI67 expression in panel L. (N) Representative immunofluorescence images showing the effect of Capn2 overexpression on KI67 expression in subcutaneous tumors derived from human pancreatic cancer organoids. (O) Bar graph showing the quantification of KI67 expression in panel N. ^*^ *P*<0.05, ^**^ *P*<0.01, ^***^ *P*<0.001.

Our previous data demonstrated that CAPN2 has a significant impact on patient prognosis. To elucidate the function of CAPN2, we knocked down CAPN2 in pancreatic-like organoids and pancreatic cancer cell lines (Supplementary Fig. S2A, B, Supplemental Digital Content 3, http://links.lww.com/JS9/D518). CAPN2 knockdown significantly inhibited goblet formation in pancreatic cancer-like organoids (Fig. 4A, B) and cell line colony formation (*P*<0.01) (Supplementary Fig. S1C, D, Supplemental Digital Content 2, http://links.lww.com/JS9/D517). Moreover, overexpression of CAPN2 significantly promoted goblet cell formation (*P*<0.01) and cell line colony formation in pancreatic cancer organoids (*P*<0.001) (Fig. 4C, D and Supplementary Fig. S1E, F, G, H, Supplemental Digital Content 2, http://links.lww.com/JS9/D517). Additionally, CAPN2 knockdown significantly reduced Ki67-positive cell numbers in the organoids, suggesting significantly reduced proliferation (*P*<0.001) (Fig. 4E, F). In contrast, CAPN2 overexpression significantly increased Ki67-positive cells in organoids, indicating significantly enhanced proliferation (*P*<0.05) (Fig. 4G, H). This demonstrates that CAPN2 plays a significant role in promoting the proliferation of pancreatic cancer organoids. Importantly, these in-vitro findings were corroborated by our in-vivo experiments. CAPN2 knockdown in nude mice significantly reduced tumor size and volume, while CAPN2 overexpression significantly promoted tumor growth (*P*<0.001) (Fig. 4I, J, K). Additionally, Ki67 staining of tumor sections confirmed a significant reduction in proliferative activity after CAPN2 knockdown (*P*<0.001) (Fig. 4L, M) and significantly increased proliferation with CAPN2 overexpression (*P*<0.01) (Fig. 4N, O). These findings indicate that the function of CAPN2 is diametrically opposed to that of METTL16, further highlighting its significant role in tumor progression and proliferation.

### METTL16 acts as a tumor growth regulator by modulating CAPN2

**Figure 5:**
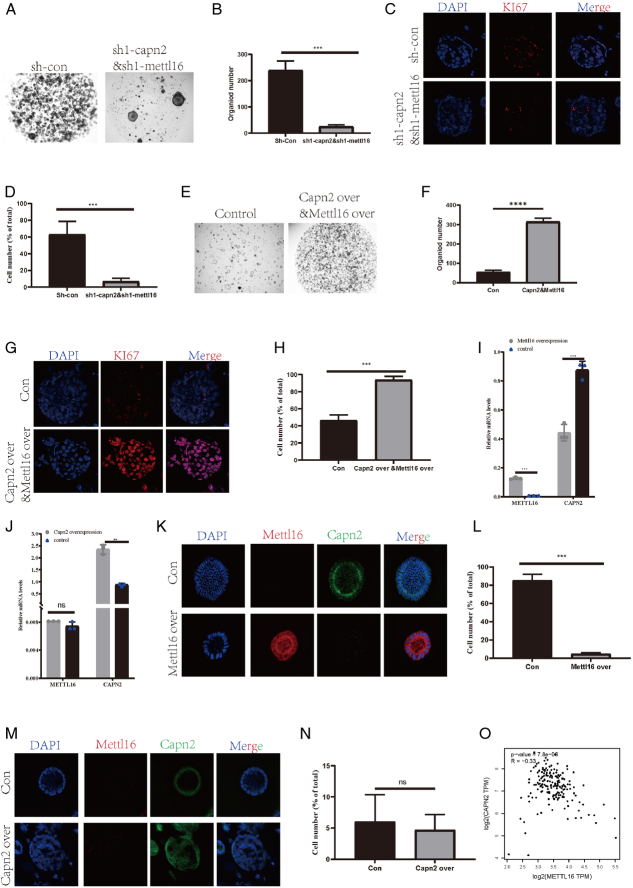
Illustrates that METTL16 can inhibit the function of CAPN2. (A) Representative images comparing the impact of CAPN2 and METTL16 knockdown on organoid formation in human pancreatic cancer-like organs. (B) Statistical bar graph of organoid formation from panel A. (C) Representative images comparing immunofluorescence of Ki67 expression in human pancreatic cancer-like organs following CAPN2 and METTL16 knockdown. (D) Statistical bar graph of organoid Ki67 expression from panel C. (E) Representative images comparing the impact of CAPN2 and METTL16 overexpression on organoid formation in human pancreatic cancer-like organs. (F) Statistical bar graph of organoid formation from panel E. (G) Representative images comparing immunofluorescence of Ki67 expression in human pancreatic cancer-like organs following CAPN2 and METTL16 overexpression. (H) Statistical bar graph of organoid Ki67 expression from panel G. (I) RT-qPCR comparison of METTL16 and CAPN2 mRNA levels after METTL16 overexpression. (J) RT-qPCR comparison of METTL16 and CAPN2 mRNA levels after METTL16 overexpression. (K) Immunofluorescence comparison of METTL16 and CAPN2 levels and colocalization after METTL16 overexpression. (L) Quantification of cell numbers expressing CAPN2. (M) Immunofluorescence comparison of METTL16 and CAPN2 levels and colocalization after CAPN2 overexpression. (N) Quantification of cell numbers expressing METTL16. (O) Correlation analysis of METTL16 and CAPN2 expression levels in the TCGA database. ^*^ *P*<0.05, ^**^ *P*<0.01, ^***^ *P*<0.001.

Previously, we noted that METTL16 is negatively correlated with the mRNA level of CAPN2. To elucidate their regulatory relationship, we investigated their upstream and downstream interactions. Concurrent knockdown of METTL16 and CAPN2 significantly decreased organoid goblet formation (Fig. 5A, B) and cell proliferation (*P*<0.001) (Fig. 5C, D). This phenotype resembled that of CAPN2 knockdown alone (Fig. 5A, B), suggesting that CAPN2 is downstream of METTL16, and METTL16 functions by regulating CAPN2. Furthermore, simultaneous overexpression of METTL16 and CAPN2 significantly increased tumor cell growth and proliferation (*P*<0.001) (Fig. 5E, F, G, H). To verify that METTL16 regulates the mRNA level of CAPN2, we observed a significant reduction in the level of CAPN2 after overexpression of METTL16 (*P*<0.001) (Fig. 5I). Interestingly, the mRNA level of METTL16 remained unchanged upon overexpression of CAPN2 (Fig. 5J), indicating that METTL16 can negatively regulate the expression of CAPN2. Since CAPN2 does not reciprocally influence METTL16 expression and no direct interaction between these two proteins was detected, these findings suggest that METTL16 regulates CAPN2 through an indirect mechanism. Immunofluorescence further confirmed our previous conclusion (Fig. 5K–N). Moreover, reanalysis of the TCGA data revealed a significant difference in the reciprocal mRNA levels between METTL16 and CAPN2 (*r*=−0.33, *P*<0.001) (Fig. 5M).

### METTL16 inhibits CAPN2 function by upregulating the transcription of MROH8

**Figure 6:**
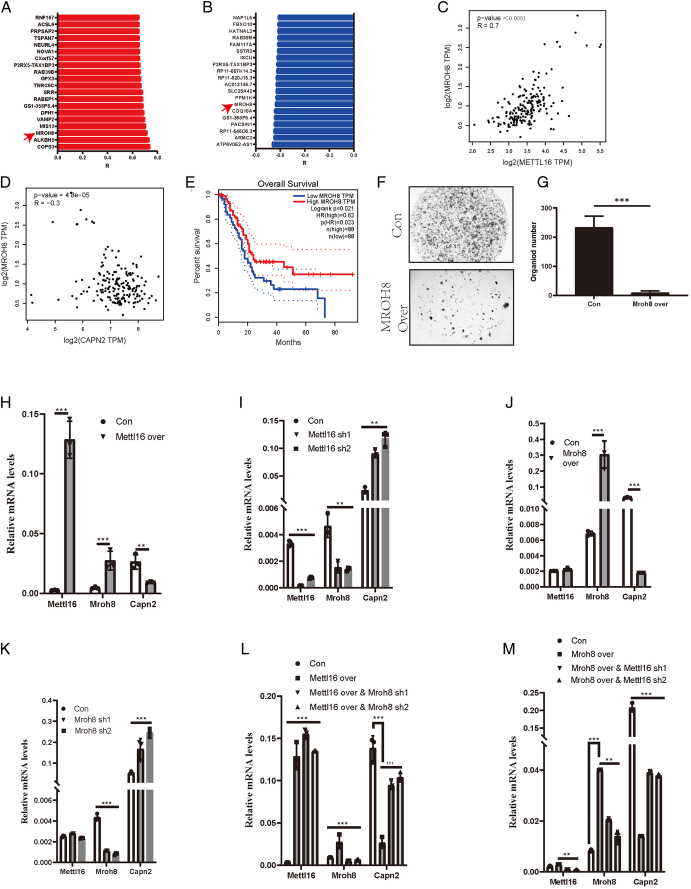
METTL16 promotes the function of MROH8. (A) Bar chart of mRNA expression levels positively correlated with METTL16 expression in TCGA database. (B) Bar chart of mRNA expression levels negatively correlated with CAPN2 expression in TCGA database. (C) Scatter plot showing the correlation between METTL16 and MROH8 expression in TCGA database. (D) Scatter plot showing the correlation between CAPN2 and MROH8 expression in TCGA database. (E) Survival analysis of high and low MROH8 mRNA expression levels in TCGA database. (F) Representative image comparing the effects of MROH8 overexpression on organ formation in human pancreatic cancer cells. (G) Bar chart of organ formation statistics in the F figure. (H) Comparison of mRNA levels of METTL16, MROH8, and CAPN2 after METTL16 overexpression using RT-qPCR. (I) Comparison of mRNA levels of METTL16, MROH8, and CAPN2 after METTL16 knockdown using RT-qPCR. (J) Comparison of mRNA levels of METTL16, MROH8, and CAPN2 after MROH8 overexpression using RT-qPCR. (K) Comparison of mRNA levels of METTL16, MROH8, and CAPN2 after MROH8 knockdown using RT-qPCR. (L) Comparison of mRNA levels of METTL16, MROH8, and CAPN2 after METTL16 overexpression and MROH8 knockdown using RT-qPCR. (M) Comparison of mRNA levels of METTL16, MROH8, and CAPN2 after MROH8 overexpression and METTL16 knockdown using RT-qPCR. ^*^ *P*<0.05, ^**^ *P*<0.01, ^***^ *P*<0.001.

Previous data showed that METTL16 can inhibit CAPN2 expression. However, as an mRNA modification writer, METTL16 typically enhances target gene expression by modifying mRNAs. This discrepancy led us to hypothesize that METTL16 affects CAPN2 through other gene expressions. To investigate this, we reanalyzed the TCGA database to identify genes positively correlated with METTL16 expression (Fig. 6A) and negatively correlated with CAPN2 expression (Fig. 6B). Fortunately, we found MROH8, a gene positively regulated by METTL16 (*r*=0.7, *P*<0.001) (Fig. 6C) and negatively regulated by CAPN2 (*r*=−0.3, *P*<0.001) (Fig. 6D). Additionally, MROH8 was associated with improved survival in pancreatic cancer patients (Fig. 6E), supporting our hypothesis. To verify the function of MROH8, we knocked down MROH8 in cell lines (Supplementary Fig. S3A, B, Supplemental Digital Content 4, http://links.lww.com/JS9/D519), which significantly promoted cell colony formation (*P*<0.001) (Supplementary Fig. S3C, D, Supplemental Digital Content 4, http://links.lww.com/JS9/D519). Conversely, overexpression of MROH8 significantly inhibited tumor cell growth (*P*<0.001) (Fig. 6F, G and Supplementary Fig. S3E–H, Supplemental Digital Content 4, http://links.lww.com/JS9/D519). Further analysis showed that METTL16 overexpression significantly increased MROH8 levels and significantly decreased CAPN2 levels (*P*<0.01) (Fig. 6H). Knocking down METTL16 significantly decreased MROH8 levels and increased CAPN2 levels (*P*<0.001) (Fig. 6I). This suggests that METTL16 can negatively regulate CAPN2 by regulating MROH8, while MROH8 can negatively regulate CAPN2. To confirm this, we overexpressed MROH8 and observed the expression of METTL16 and CAPN2. Interestingly, METTL16 levels remained unchanged, while CAPN2 levels decreased significantly (*P*<0.001) (Fig. 6J), indicating that MROH8 negatively regulates CAPN2. Subsequent knockdown of MROH8 confirmed this conclusion (Fig. 6K). To demonstrate that METTL16 regulates CAPN2 through MROH8, we knocked down MROH8 after overexpression of METTL16. Compared with the reduction observed with METTL16 overexpression alone, the levels of CAPN2 significantly increased (*P*<0.001) (Fig. 6L). Next, we knocked down METTL16 after overexpressing MROH8. Compared to the reduction in CAPN2 expression observed with MROH8 overexpression alone, CAPN2 expression significantly increased after METTL16 knockdown (*P*<0.001) (Fig. 6M). This further supports the notion that CAPN2 is regulated by MROH8.

### The m6A recognition gene YTHDC2 and the m6A writer gene METTL16 collaborate to regulate MROH8, thereby enhancing its mRNA stability

**Figure 7:**
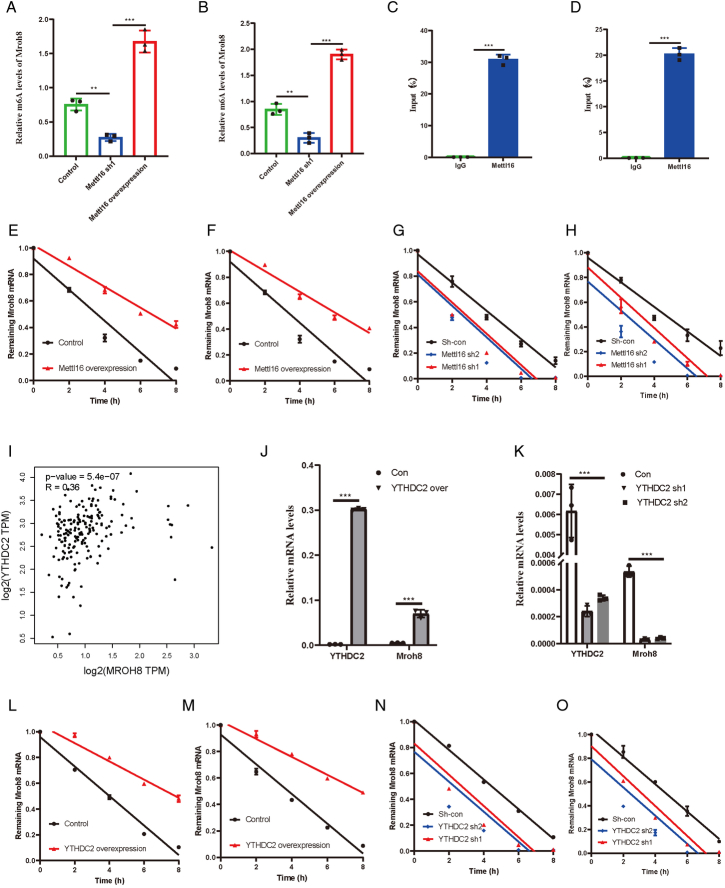
METTL16 and YTHDC2 promote the mRNA stability of MROH8 through m6A modification. (A) Comparison of the related m6A levels of MROH8 after METTL16 overexpression and knockdown in human pancreatic cancer organoids. (B) Comparison of the related m6A levels of MROH8 after METTL16 overexpression and knockdown in PANC1 pancreatic cancer cell line. (C) Comparison of METTL16 levels related to MROH8 in pancreatic cancer organoids using METTL16 RIP-qPCR. (D) Comparison of METTL16 levels related to MROH8 in PANC1 pancreatic cancer cell line using METTL16 RIP-qPCR. (E) mRNA stability of MROH8 after METTL16 overexpression in human pancreatic cancer organoids. (F) mRNA stability of MROH8 after METTL16 overexpression in PANC1 pancreatic cancer cell line. (G) mRNA stability of MROH8 after METTL16 knockdown in human pancreatic cancer organoids. (H) mRNA stability of MROH8 after METTL16 knockdown in PANC1 pancreatic cancer cell line. (I) Correlation between m6A reader YTHDC2 and MROH8 mRNA expression levels in TCGA database. (J) Comparison of MROH8 mRNA levels after YTHDC2 overexpression using RT-qPCR. (K) Comparison of MROH8 mRNA levels after YTHDC2 knockdown using RT-qPCR. (L) mRNA stability of MROH8 after YTHDC2 overexpression in human pancreatic cancer organoids. (M) mRNA stability of MROH8 after YTHDC2 overexpression in PANC1 pancreatic cancer cell line. (N) mRNA stability of MROH8 after YTHDC2 knockdown in human pancreatic cancer organoids. (O) mRNA stability of MROH8 after YTHDC2 knockdown in PANC1 pancreatic cancer cell line. ^*^ *P*<0.05, ^**^ *P*<0.01, ^***^ *P*<0.001.

Previous experiments indicated that METTL16 can regulate MROH8 mRNA levels. METTL16 is known as an m6A writer gene, capable of stabilizing target gene transcription by adding m6A modifications. Based on this, we hypothesized that METTL16 also acts on MROH8 through m6A modifications. To confirm this, we performed gene-specific m6A qPCR, showing that METTL16 overexpression significantly increased m6A levels in MROH8 (*P*<0.01), while knockdown significantly reduced its abundance (*P*<0.001) (Fig. 7A, B). Additionally, our METTL16 RIP-qPCR data demonstrated strong binding between METTL16 protein and MROH8 transcripts in pancreatic cancer cells (Fig. 7C, D). Furthermore, we verified that METTL16 overexpression promoted MROH8 mRNA stability, while knockdown reduced it (Fig. 7E–H). Since m6A writers alone are not sufficient, we investigated m6A readers. Analyzing TCGA data revealed that YTHDC2 had a positive correlation with MROH8 (*r*=0.36, *P*<0.001) (Fig. 7I). YTHDC2 knockdown significantly increased the expression of MROH8 (*P*<0.001) (Fig. 7J) while YTHDC2 overexpression significantly decreased it (*P*<0.001) (Fig. 7K) indicating that YTHDC2 plays a role in affecting mRNA stability by regulating MROH8 degradation rates. Specifically, YTHDC2 overexpression significantly prolonged the half-life of MROH8 mRNA (Fig. 7L, M), while YTHDC2 knockdown accelerated its degradation (Fig. 7N, O), further demonstrating its role in mRNA stability regulation.

### MROH8 modulates the transcriptional regulator TBP

**Figure 8:**
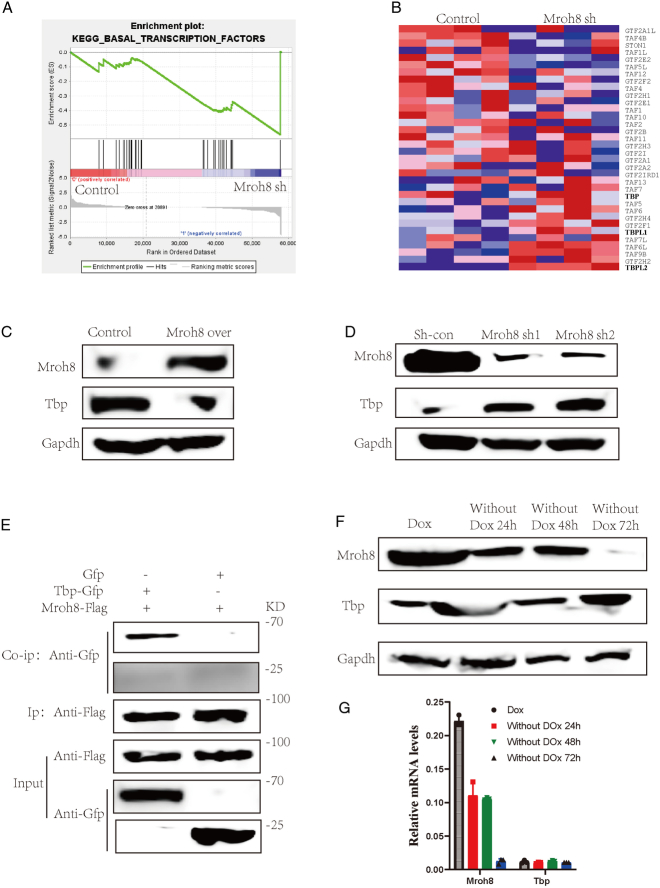
MROH8 binds to TBP causing its protein degradation. (A) Signal pathway analysis comparing MROH8 knockdown and control group. (B) Differential protein analysis heatmap of MROH8 knockdown and control group through proteomic analysis. (C) Western blot comparison of TBP and MROH8 expression levels after MROH8 overexpression. (D) Western blot comparison of TBP and MROH8 expression levels after MROH8 knockdown. (E) CO-IP comparison of MROH8 and TBP protein interactions. (F) Western blot comparison of MROH8 and TBP protein levels at different time points after doxycycline withdrawal. (G) RT-qPCR comparison of MROH8 and TBP mRNA levels at different time points after doxycycline withdrawal.

Although we have shown that MROH8 can be regulated by m6A modifications, its role in regulating CAPN2 remains a mystery without relevant reports in the current literature. To investigate, we first analyzed proteomics data of MROH8 high and low expression. Enrichment analysis revealed that MROH8 negatively regulated the levels of transcription factors (Fig. 8A). Differential protein analysis showed that high MROH8 levels correlated with significantly lower TBP levels (Fig. 8B). We hypothesized that MROH8 regulates TBP at the protein level. Using Western blotting, we confirmed that TBP protein levels decreased with MROH8 overexpression and increased with MROH8 knockdown (Fig. 8C, D). Co-immunoprecipitation experiments demonstrated that MROH8 binds to TBP protein (Fig. 8E). We speculated that MROH8 influences TBP protein degradation after binding. Inducing MROH8 overexpression and withdrawing the inducer doxycycline over time showed a decrease in MROH8 levels and an increase in TBP levels (Fig. 8F). Interestingly, TBP transcript levels did not change with decreasing MROH8 (Fig. 8G), indicating that MROH8 affects TBP protein levels.

### TBP enhances CAPN2 transcription by binding to the CAPN2 promoter

**Figure 9:**
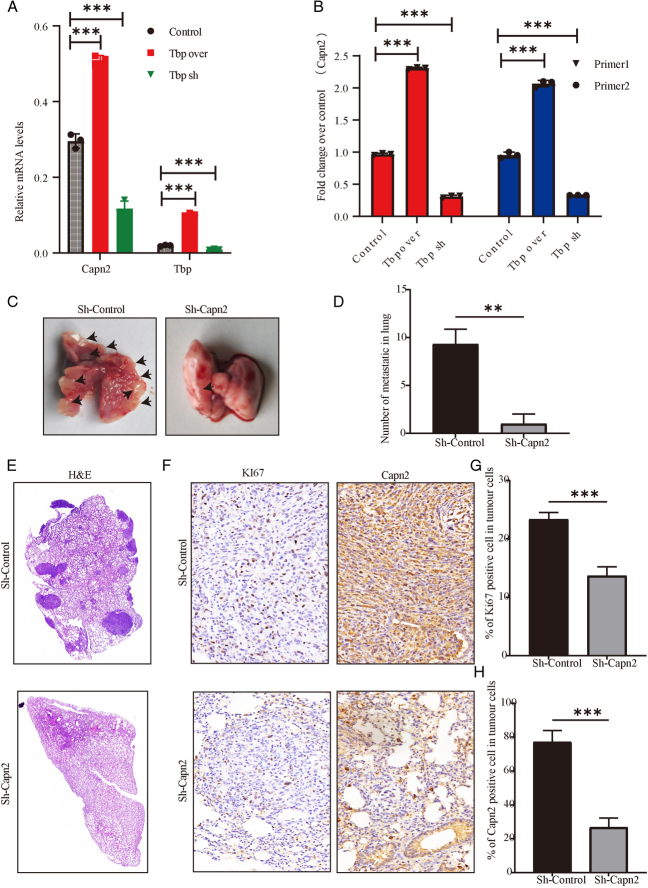
TBP affects CAPN2 translation by binding to its promoter. (A) RT-qPCR comparison of CAPN2 mRNA levels after TBP knockdown and overexpression. (B) Chip comparison of TBP overexpression and knockdown with CAPN2 promoter binding. (C) Representative image of the effects of CAPN2 knockdown on lung metastasis in mice via tail vein injection. (D) Bar chart of statistical analysis of lung metastatic foci in C figure. (E) Representative images of H&E staining of lung metastatic tumors in C figure using immunohistochemistry. (F) Representative images of Ki67 and CAPN2 staining of lung metastatic tumors in C figure using immunohistochemistry. (G) Bar chart of statistical analysis of Ki67-positive cells in F figure. (H) Bar chart of statistical analysis of CAPN2-positive cells in F figure. ^*^ *P*<0.05, ^**^ *P*<0.01, ^***^ *P*<0.001.

Previously, we demonstrated that MROH8 influences TBP function by affecting TBP protein levels. Given TBP’s role as a crucial transcription factor and MROH8’s ability to inhibit CAPN2 levels, we investigated whether MROH8 could inhibit TBP, thus impacting CAPN2 transcription. To test this, we observed CAPN2 mRNA levels after manipulating TBP expression and found that TBP significantly promoted CAPN2 mRNA levels (*P*<0.001) (Fig. 9A). Furthermore, we explored whether TBP functions by binding to the CAPN2 promoter using CHIP analysis. We observed that the binding of TBP to the CAPN2 promoter was significantly increased upon TBP overexpression, and conversely decreased upon TBP knockdown (*P*<0.001) (Fig. 9B), indicating that TBP can modulate the transcriptional level of CAPN2.

### CAPN2 significantly promotes tumor metastasis

Literature reports suggest that CAPN2 not only affects tumor cell proliferation but also promotes tumor metastasis. To investigate this, we assessed lung metastasis by knocking down CAPN2 and injecting cells into the mouse tail vein. We observed a significant reduction in the number of lung metastases after CAPN2 knockdown (*P*<0.01) (Fig. 9C, D). Additionally, the activity of tumor cells in lung metastases was significantly reduced after CAPN2 knockdown (*P*<0.001) (Fig. 9E–H), indicating that CAPN2 plays a critical role in metastasis and the growth of tumor cells post-metastasis.

## Discussion

At the time of diagnosis, most pancreatic cancers are too advanced for surgery to be an option at the time of diagnosis, typically being either locally advanced (stage III) or having metastasized to other parts of the body (stage IV). This is due to the fact that early-stage pancreatic cancer may be asymptomatic, or symptoms may be vague and difficult to detect^18^. Thus, understanding the mechanisms that drive pancreatic cancer metastasis is critical for developing new therapeutic strategies. m6A is a critical post-transcriptional modification that plays a key role in the development and progression of various cancers^19^. Aberrations in m6A have been linked to tumorigenesis, metastasis, and cancer progression^20^. In pancreatic cancer, m6A methylation influences key pathways involved in cell proliferation and metastasis, underscoring its role in tumor development^21^. To further explore the impact of m6A genes on pancreatic cancer prognosis, we conducted transcriptomic analyses and identified METTL3 and METTL16 as the key m6A ‘writer’ genes associated with better patient outcomes. Our correlation analysis revealed several core genes, including CAPN2, CHMP2B, ITGA3, ITGA6, ITPR1, and RAC1, which were significantly linked to patient prognosis and m6A expression. These findings suggest that the METTL16 and CAPN2 axis may play a particularly important role in pancreatic cancer progression, especially as we observed higher expression of CAPN2 in advanced-stage tumors and in tumor cell lines compared to normal pancreatic cells. This highlights the potential prognostic and therapeutic relevance of this regulatory network in pancreatic cancer.

In this context, our study focused on METTL16, an m6A writer, which has been shown to play a dual role in cancers. In some cases, it promotes tumor progression by stabilizing oncogenic mRNAs^22^, while in pancreatic cancer, it has been linked to tumor suppression by regulating key tumor suppressor pathways^23^. Its ability to modulate mRNA stability and translation depends on the cancer type and context^24^. CAPN2, a calcium-dependent cysteine protease, has been implicated in cancer progression, including pancreatic cancer. It promotes epithelial–mesenchymal transition (EMT) and metastasis via the Wnt/β–catenin pathway and its overexpression is linked to poor prognosis^25,26^. Emerging evidence suggests that m6A regulators, such as METTL16, may interact with CAPN2 to influence tumor progression through post-transcriptional mechanisms^27^. Our study found that METTL16 suppresses pancreatic cancer progression by downregulating CAPN2 via m6A-mediated regulation. This aligns with previous findings that METTL16 can act as a tumor suppressor in some cancers, while CAPN2 drives tumor growth. Our results support the role of METTL16–CAPN2 regulation in pancreatic cancer progression and highlight this axis as a potential therapeutic target^23,25,27^.

TATA-binding protein (TBP) is a crucial transcription factor that binds to the TATA box sequence in the promoter regions of genes, initiating transcription by facilitating the assembly of the transcription machinery^28^. In some eukaryotic gene promoters, this DNA sequence is located ~30 base pairs upstream of the transcription start site^29^. TBP is a member of the small gene family of TBP-associated factors. The first TBP-associated factor (TRF/TRF1) was found in the fruit fly *Drosophila* but appears to be present only in *Drosophila* or insects. Subsequently, TBPL1/TRF2 was found in the genomes of many postnatal animals, while the vertebrate genome encodes a third vertebrate family member, TBPL2/TRF3. TBP can be replaced by one of these TBP-associated factors at a specific cell type or a specific promoter, and some of these interact with TBP similarly to the TATA box^30^. TBP plays a significant role in regulating gene expression and has been implicated in various cellular processes, including those involved in cancer progression^31^. Additionally, recent findings have linked TBP to m6A modifications, showing that TBP can influence m6A methylation processes by promoting the expression of key m6A writers such as METTL3, which enhances RNA stability and translation in cancer cells^32^. For instance, TBP binding to the METTL3 promoter has been shown to increase m6A methylation, impacting cancer cell metabolism and proliferation^33^. However, TBP’s role in m6A processes remains underexplored, especially regarding its interaction with other regulatory proteins in different cancer types. Therefore, understanding TBP’s broader role in m6A-related regulation may provide new insights into its contribution to cancer progression.

**Figure 10:**
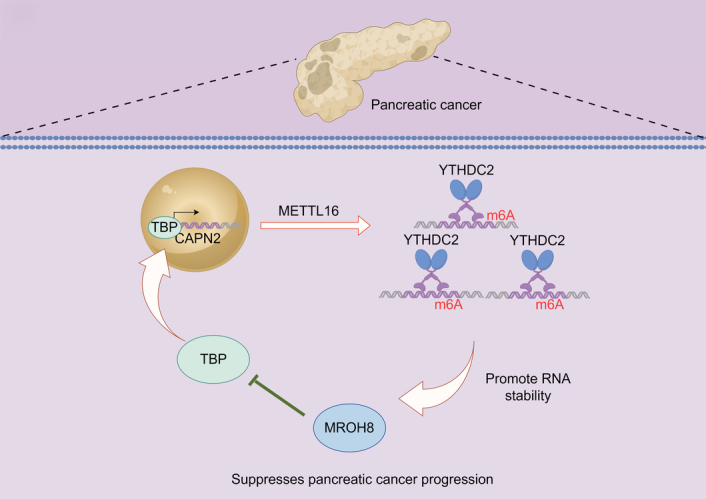
Mechanism overview diagram.

Our study identified MROH8 as a novel regulator interacting with TBP, influencing its stability and function in the context of m6A methylation. The discovery of MROH8’s involvement in TBP regulation adds a new dimension to the understanding of TBP function in cancer. MROH8 was identified as part of a gene expression signature related to temozolomide sensitivity in glioblastomas, providing potential prognostic value and therapeutic guidance for patient survival^34^. We confirmed the physical interaction between MROH8 and TBP through Co-IP, demonstrating a direct binding between these two proteins. This interaction appears to influence TBP protein stability, as MROH8 overexpression promotes TBP degradation, while MROH8 knockdown increases TBP levels. Furthermore, METTL16 is able to maintain MROH8 expression through the action of m6A. This finding is consistent with reports highlighting TBP’s role in m6A-related transcriptional regulation and adds a new layer of understanding to how MROH8 may modulate disease progression through post-translational regulation of TBP. These insights suggest a complex interplay between TBP, m6A modification, and disease progression, warranting further investigation. However, despite these promising findings, it is important to acknowledge that the association of the genes identified in this study with pancreatic cancer prognosis is based solely on predictive analyses from databases, and the clinical relevance of the findings needs to be validated through further studies. Additionally, our findings were validated only in the pancreatic cancer PDOX animal model and cell lines, so further research is necessary to confirm the clinical applicability of these results in human patients. In conclusion, this study uncovers a novel regulatory pathway in pancreatic cancer involving METTL16, MROH8, TBP, and m6A modifications. The mechanism is summarized in Figure 10. We found that METTL16 enhances m6A modifications and regulates TBP, which activates CAPN2 expression, while MROH8 inhibits TBP by promoting its degradation, thereby reducing CAPN2 transcription. Elevated CAPN2 levels are linked to advanced tumor stages and poor prognosis, making the METTL16–MROH8–TBP–CAPN2 axis a potential therapeutic target for pancreatic cancer.

## Ethical approval

The experimental protocol was approved by the Clinical Research and Animal Experimentation Ethics Committee of the Affiliated Hospital of Youjiang Medical College of Nationalities [SYXK Gui 2017-0004, Ethics No. 2022052802]. All patients provided written informed consent. The animal experiments were performed under the approval of the committee on the Ethics of Animal Experiments of the Affiliated Hospital of Youjiang Medical College of Nationalities.

## Consent

None.

## Source of funding

1. Natural Science Fund Project of Guangxi Province, China (No. 2020GXNSFAA297170).

2. Project funded by China Postdoctoral Science Foundation (No. 2022MD723766, 2023MD734159).

3. National Natural Science Foundation Regional Science Foundation Project, China (No. 82260532).

4. National Natural Science Foundation of China (grant no. 82360837).

## Author contribution

H.L., J.M., C.L., and Z.W.: designed the research; T.Y., C.W., X.Y., and J.L.: performed the research; C.L., F.Q., W.Y., Y.Y., J.L., and D.N.: analyzed the data and wrote the paper. All authors read and approved the final manuscript.

## Conflicts of interest disclosure

The authors declare no conflicts of interest.

## Research registration unique identifying number (UIN)

None.

## Guarantor

Huafu Li, MD; e-mail: Huafu.li@ic.ac.uk; Chunying Luo, MD; e-mail: lcy2005@ymun.edu.cn; Jian Ma, MD; e-mail: majian7@mail3.sysu.edu.cn; Zhongheng Wei, MD; e-mail: zhonghengwei1@163.com.

## Data availability statement

The datasets from the current study are available from the corresponding author on reasonable request.

## Provenance and peer review

Not commissioned, externally peer-reviewed.

## Supplementary Material

SUPPLEMENTARY MATERIAL
