# Supplementary figures and images for: METTL16 inhibits pancreatic cancer proliferation and metastasis by promoting MROH8 RNA stability and inhibiting CAPN2 expression – experimental studies
Source: Int J Surg. 2024 Oct 22;110(12):7701–19. doi: 10.1097/JS9.0000000000002116 (PMC11634154; doi:10.1097/JS9.0000000000002116)

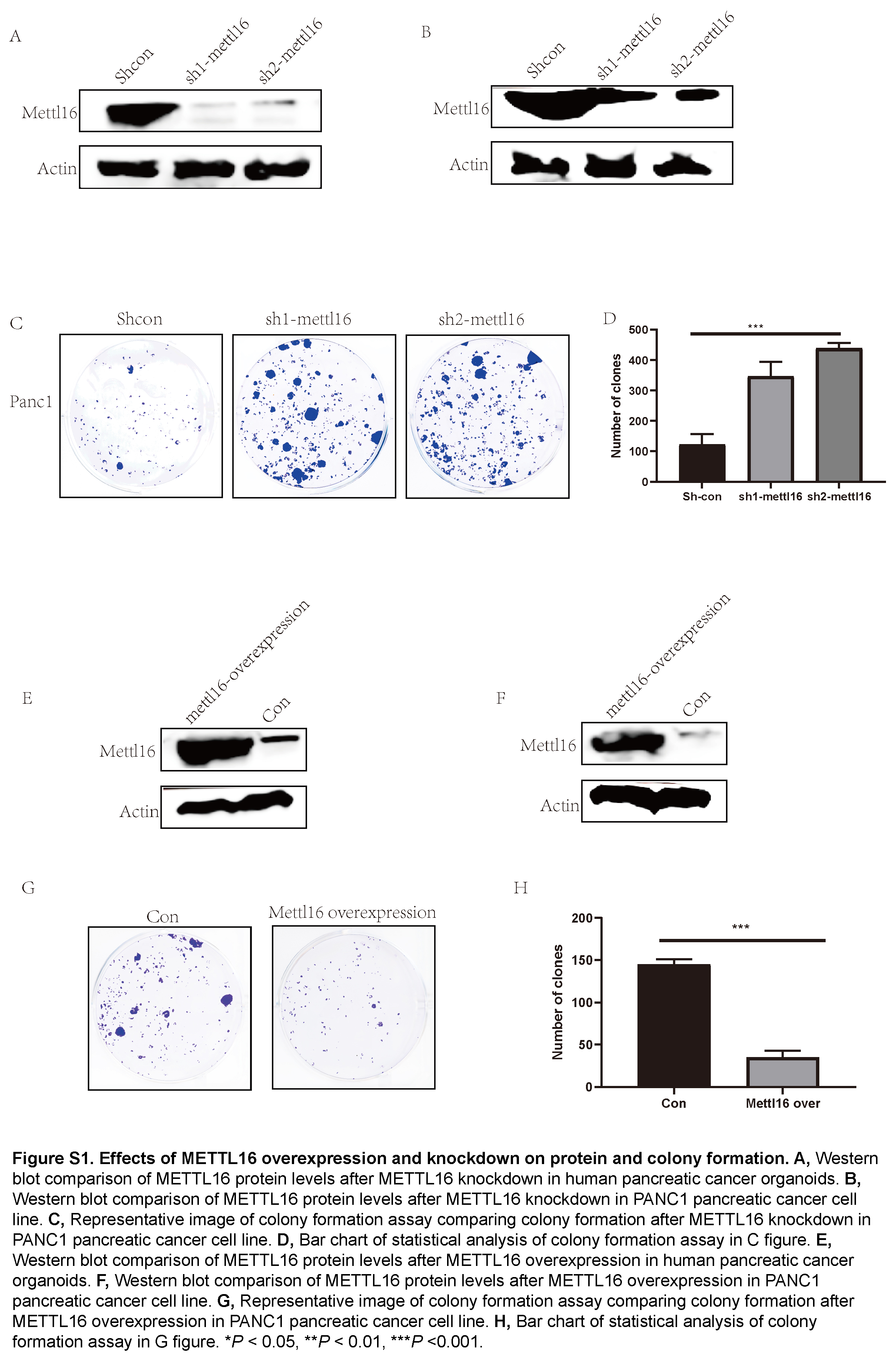

Supplement: SUPPLEMENTARY MATERIAL [file js9-110-7701-s002.tif]

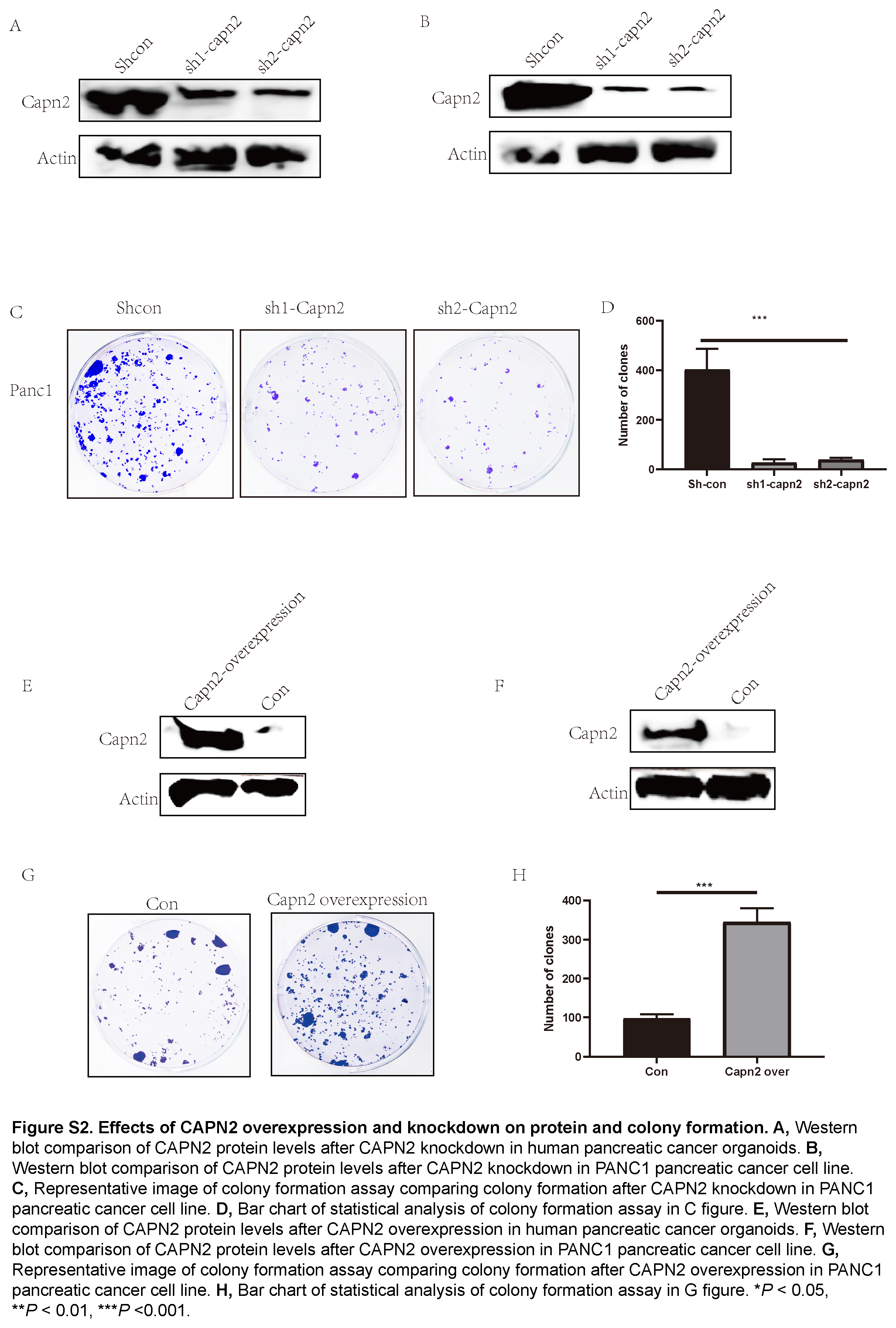

Supplement: SUPPLEMENTARY MATERIAL [file js9-110-7701-s003.tif]

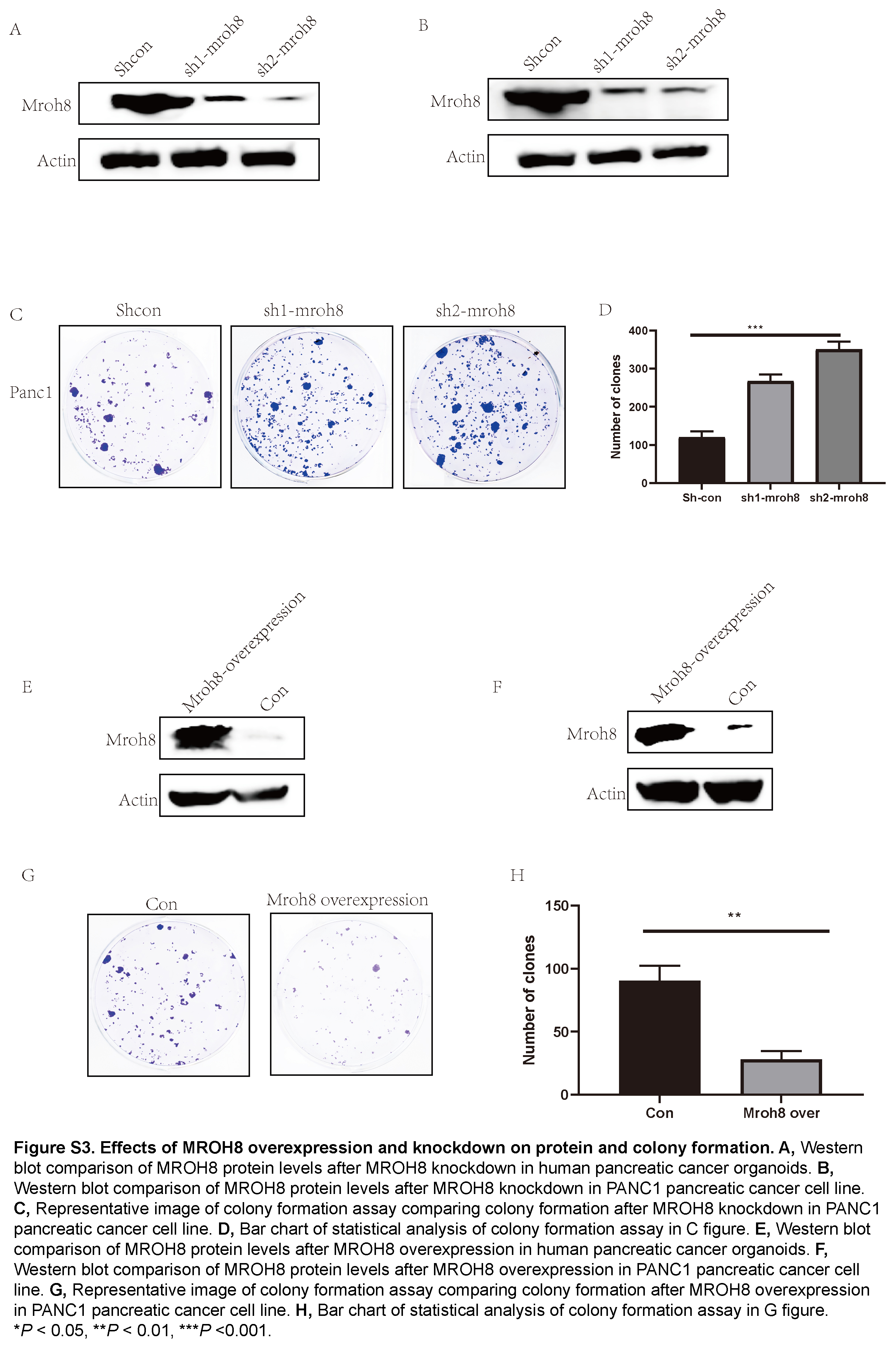

Supplement: SUPPLEMENTARY MATERIAL [file js9-110-7701-s004.tif]
